# Disturbance of the Warburg effect by dichloroacetate and niclosamide suppresses the growth of different sub-types of malignant pleural mesothelioma *in vitro* and *in vivo*

**DOI:** 10.3389/fphar.2022.1020343

**Published:** 2022-10-11

**Authors:** Sze-Kwan Lam, Sheng Yan, Joyce Sze-Man Lam, Yuqian Feng, Mahjabin Khan, Caoyang Chen, Frankie Chi-Fat Ko, James Chung-Man Ho

**Affiliations:** Division of Respiratory Medicine, Department of Medicine, Queen Mary Hospital, The University of Hong Kong, Pokfulam, Hong Kong SAR, China

**Keywords:** malignant pleural mesothelioma, warburg effect, dichloroacetate, niclosamide, apoptosis, xenografts

## Abstract

**Background:** Inhalation of asbestos fibers is the most common cause of malignant pleural mesothelioma (MPM). In 2004, the United States Food and Drug Administration approved a combination of cisplatin with pemetrexed to treat unresectable MPM. Nonetheless novel treatment is urgently needed. The objective of this study is to report the combination effect of dichloroacetate (DCA) or niclosamide (Nic) Nic in MPM.

**Materials and methods:** The effect of a combination of DCA and Nic was studied using a panel of MPM cell lines (H28, MSTO-211H, H226, H2052, and H2452). Cell viability was monitored by MTT assay. Glycolysis, oxidative phosphorylation, glucose, glycogen, pyruvate, lactate, citrate, succinate and ATP levels were determined by corresponding ELISA. Apoptosis, mitochondrial transmembrane potential, cell cycle analysis, hydrogen peroxide and superoxide were investigated by flow cytometry. Cell migration and colony formation were investigated by transwell migration and colony formation assays respectively. The *in vivo* effect was confirmed using 211H and H226 nude mice xenograft models.

**Results and conclusion:** Cell viability was reduced. Disturbance of glycolysis and/or oxidative phosphorylation resulted in downregulation of glycogen, citrate and succinate. DCA and/or Nic increased apoptosis, mitochondrial transmembrane depolarization, G2/M arrest and reactive oxygen species. Moreover, DCA and/or Nic suppressed cell migration and colony formation. Furthermore, a better initial tumor suppressive effect was induced by the DCA/Nic combination compared with either drug alone in both 211H and H226 xenograft models. In H226 xenografts, DCA/Nic increased median survival of mice compared with single treatment. Single drug and/or a combination disturbed the Warburg effect and activated apoptosis, and inhibition of migration and proliferation *in vivo*. In conclusion, dichloroacetate and/or niclosamide showed a tumor suppressive effect in MPM *in vitro* and *in vivo,* partially mediated by disturbance of glycolysis/oxidative phosphorylation, apoptosis, ROS production, G2/M arrest, and suppression of migration and proliferation.

## 1 Introduction

Mesothelioma is a rare malignant tumor that grows on the mesothelial surface of coelomic cavities including the tunica vaginalis, pericardium, pleura and peritoneum. Malignant pleural mesothelioma (MPM) is the most common form of this malignancy. Asbestos is a set of silicate minerals found in nature. Inhalational exposure to this carcinogenic substance is the major cause of MPM (Ortega-Guerrero et al., 2014). Asbestos is commonly used in construction and industrial materials, making MPM an occupational disease that predominantly affects males. Pemetrexed in combination with cisplatin has become the cornerstone of chemotherapy in the clinical management of advanced MPM (Vogelzang et al., 2003).

The Warburg effect is the phenomenon of aerobic glycolysis in cancer cells including mesothelioma (Zhang et al., 2009a) that leads to preferential conversion of glucose to lactate, even in aerobic conditions (Sun et al., 2018). The Warburg effect provides NADPH and other metabolic intermediates, which are strong reducing agents and essential biomass, for cell proliferation. Theoretically, inhibition of the Warburg effect should be an effective and universal anticancer strategy. The key intermediates in glycolysis and Krebs cycle are shown in Supplementary Figure S1.

Inhibition of the Warburg effect has been studied extensively in different cancer types. Nonetheless much less is known about disruption of the Warburg effect in MPM. There are only a few reports of the Warburg effect in mesothelioma: inhibition by citrate (phosphofructokinase inhibitor) with cisplatin (Zhang et al., 2009b), 3-Bromopyruvate (hexokinase II Inhibitor II) with cisplatin (Zhang et al., 2009a; Icard et al., 2012) and metformin (mitochondrial respiratory complex I inhibitor) with nutlin-3a (wild-type *p53* inhibitor) (Shimazu et al., 2017).

Dichloroacetate is a prescribed drug for lactic acidosis. The anticancer effect of DCA has been shown in breast (Xintaropoulou et al., 2015), cervical (Li et al., 2017a), colorectal (Tong et al., 2011) and lung cancers (Lu et al., 2018) as well as glioma (Duan et al., 2013), melanoma (Abildgaard et al., 2017), lymphoma (Kumar et al., 2012), and leukemia (Voltan et al., 2016) *via* various mechanisms including inhibition of PDK1–4, apoptosis and cell cycle arrest. Niclosamide is an oral anthelminthic drug used to treat parasitic infestations. It has been demonstrated that niclosamide can suppress tumor growth in different cancer types, e.g., adrenocortical carcinoma (Khan et al., 2016), glioma (Wieland et al., 2013), and leukemia (Chae et al., 2018), as well as head and neck (Wang et al., 2017), breast (Yin et al., 2016), lung (Xiang et al., 2017), ovarian (Arend et al., 2016), prostate (Lu et al., 2011) and renal (Yu et al., 2018) cancers *via* different mechanisms, including inhibition of Wnt, Notch, mTOR, and NF-κB signaling. In the preliminary experiment, we selected 3 reagents related to the Warburg effect to screen for anticancer activity in MPM cell lines: metformin, dichloroacetate and niclosamide. Synergism was observed when dichloroacetate was combined with niclosamide. The mechanisms of action are disclosed.

## 2 Materials and methods

### 2.1 Cell lines and reagents

A panel of 5 mesothelioma cell lines (NCI-H28 (sarcomatoid), MSTO-211H (biphasic), NCI-H226 (epithelioid), NCI-H2052 (sarcomatoid) and NCI-H2452 (epithelioid)) was purchased and authenticated (American Type Culture Collection, Manassas, VA, United States). Cells were incubated in RPMI-1640 medium (Gibco®, Life Technologies, Carlsbad, California, United States) enriched with 10% fetal bovine serum (FBS) (Gibco®) in a humidified atmosphere of 5% CO_2_ at 37°C (Lam et al., 2017).

### 2.2 Sodium dichloroacetate and niclosamide

Sodium dichloroacetate (DCA) and niclosamide (Nic) were purchased from Sigma-Aldrich.

### 2.3 Study of protein expression with Western blot, immunohistochemistry and immunofluorescence staining

Western blot was performed as previously described (Lam et al., 2020). Specific primary antibodies [mouse monoclonal anti-human β-actin (Sigma-Aldrich); anti-PCNA, anti-PARP (Santa Cruz Biotechnology, Inc., Santa Cruz, California, United States of America); anti-PFKP, anti-LDHB, anti-Bcl-2, anti-survivin, anti-XIAP, anti-AKT, anti-CDK2, anti-CDK4, anti-CDK7, anti-Cyclin D2, H, anti-CDK4, anti-N-cadherin, anti-β-catenin (Cell Signaling Technology, Danvers, Massachusetts, United States of America); and corresponding horseradish peroxidase (HRP)-conjugated secondary (Cell Signaling Technology)] were purchased. An enhanced chemiluminescence (ECL) kit (GE Healthcare) was used to detect protein expression. Beta-actin was selected as a reference protein (Lam et al., 2020).

### 2.4 Cell viability assay and colony formation assay

Briefly, cells (5000/well) were treated with different concentrations of DCA and/or Nic while medium only served as a negative control. Absorbance (595 nm) was measured using a microplate reader Fluo Star Optima (Bmg Labtec GmbH, Ortenberg, Germany) (Lam et al., 2017). Colony formation assay was carried out as previously reported (Iwanaga et al., 2022).

### 2.5 Detection of metabolites in glycolysis and krebs cycle

Glycolysis (Cayman ^#^600450), oxygen consumption rate (Cayman 3600800), L-lactate (Cayman ^#^700510), glucose uptake (Cayman ^#^600470), ATP (Cayman ^#^700410), and pyruvate (Cayman ^#^700470), glucose (Cayman ^#^10009582), citrate (BioAssay system, ECIT-100) and succinate (BioAssay system, ESNT-100) levels were determined according to the manufacturers’ instructions.

### 2.6 Detection of apoptosis

Apoptosis and mitochondrial membrane depolarization were determined by annexin-V binding assay and JC-1 staining assay respectively, as previously reported (Lam et al., 2016).

### 2.7 Migration and invasion assays

For migration assay, 5000 cells were suspended in 250 μl plain medium and seeded in the upper chamber while 750 μl medium containing 10% FBS was placed in the lower chamber. DCA and/or Nic were added to the upper chamber and incubated for 24 h. Cells were fixed with 4% formaldehyde for 30 min and stained with crystal violet for 2 h. Cells on the inner part of the upper chamber were removed. Photos were captured using a Nikon Ni-U fluorescence microscope (Nikon, Tokyo, Japan) equipped with a camera/detector Diagnostic Instrument RT3 Slider (Meyer Instruments, Houston, United States).

For invasion assay, a layer of Matrigel was coated on the upper chamber before adding cells. The number of cells added was 50000 for H28 cells and 500,000 for other cell lines.

### 2.8 Tumor growth inhibition *in vivo*

The 211H and H226 xenograft models were created by subcutaneous injection of 10^7^ corresponding cells in PBS into the upper back of 40 nude mice (female, 4-6-weeks-old, 10–14 g, BALB/cAnN-nu, Charles River Laboratories, Wilmington, United States). Mice were randomized to one of 4 groups after tumor growth was established. Nic was dissolved in PBS containing 10% Kolliphor® EL (Sigma-Aldrich). The mixture was sonicated for 30 min and sterilized by filtration. Solvent (control), DCA, Nic and a combination of DCA and Nic were administered intraperitoneally and daily. Tumor dimension (using standard calipers) and body weight of mice were measured twice a week and tumor volume calculated [volume = length x width x width)/2]. For humane reasons, mice were sacrificed when tumor volume reached 600 mm^3^. Tumor xenografts were collected. The study protocol was approved by the institutional Animal Ethics Committee (approval reference number: CULATR 5170-19), and standard humane endpoints for animal research were applied (Lam et al., 2020).

### 2.9 Statistical analysis

Experiments were repeated at least three times and data analyzed. Student’s two-tailed *t*-test was used for comparison of pairs. The difference between groups (more than two groups) was analyzed using variance analysis (ANOVA) by Prism (GraphPad Software, La Jolla, Southern California, United States). A *p*-value < 0.05 was considered statistically significant (*: *p* < 0.05, **: *p* < 0.01, ***: *p* < 0.001).

## 3 Results

### 3.1 Basal expression of phosphofructokinase platelet and lactate dehydrogenase B

**FIGURE 1:**
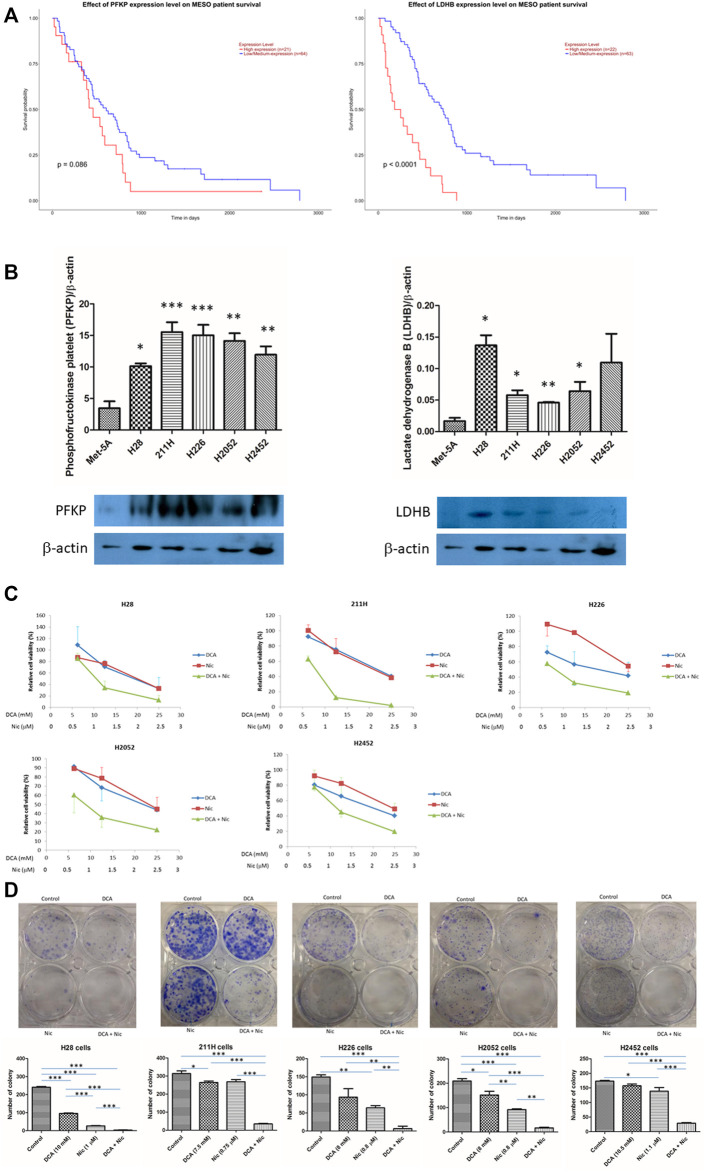
Phosphofructokinase platelet (PFKP) and lactate dehydrogenase B (LDHB) in mesothelioma. And synergism in DCA/Nic combination in MPM cell lines **(A)** Mesothelioma patients with lower expression of PFKP or LDHB have a longer median survival. **(B)** The expression of PFKP and LDHB is higher in MPM cell lines than normal mesothelial cells Met5A. **(C)** DCA or Nic reduced cell viability in a dose-dependent fashion. Synergistic effect was observed when DCA and Nic were combined. **(D)** Synergistic inhibitory effect in colony formation was observed when DCA and Nic were combined.

The expression of phosphofructokinase platelet (PFKP) and lactate dehydrogenase B (LDHB) was correlated with mesothelioma patient survival (data generated by The Cancer Genome Atlas (TCGA) research network: https://www.cancer.gov/tcga). Higher expression of PFKB and LDHB resulted in lower median survival (*p* = 0.086 and <0.0001 respectively) (Figure 1A). The expression of PFKP in MPM cell lines was about 3–5 times higher than that of normal mesothelial cell line Met5A. On the contrary, LDHB expression in cancer cell lines was 2–7 times higher than that in Met5A cells, except in H2452 cells that had larger variability (Figure 1B).

### 3.2 Reduction in cell viability

DCA or Nic alone reduced cell viability in a dose-dependent manner. When H28, 211H, H226, H2052, and H2452 cells were treated with DCA or Nic for 72 h, the IC_50_ value was 20, 21, 20, 23 and 25 mM or 2, 2, 3, 3 and 2.5 μM respectively. Furthermore, combination of DCA and Nic demonstrated a synergistic anti-cancer effect in all 5 cell lines. The IC_50_ value for DCA/Nic combination was decreased to 10, 7.5, 8, 8 and 10.5 or 1, 0.75, 0.8, 0.8 and 1.1 respectively (Figure 1C, Supplementary Table S1). DCA/Nic combination synergistically inhibited colony formation in all cell lines (Figure 1D).

### 3.3 Glycolysis and citric acid cycle

The short-term effects of DCA and/or Nic were investigated: extracellular lactate (glycolysis), oxygen consumption (oxidative phosphorylation), glucose uptake, intracellular level of glucose, glycogen, pyruvate, lactate, citrate, succinate and ATP were evaluated. There was no significant cell death during 4 h of treatment.

#### 3.3.1 Extracellular lactate (glycolysis)

**FIGURE 2:**
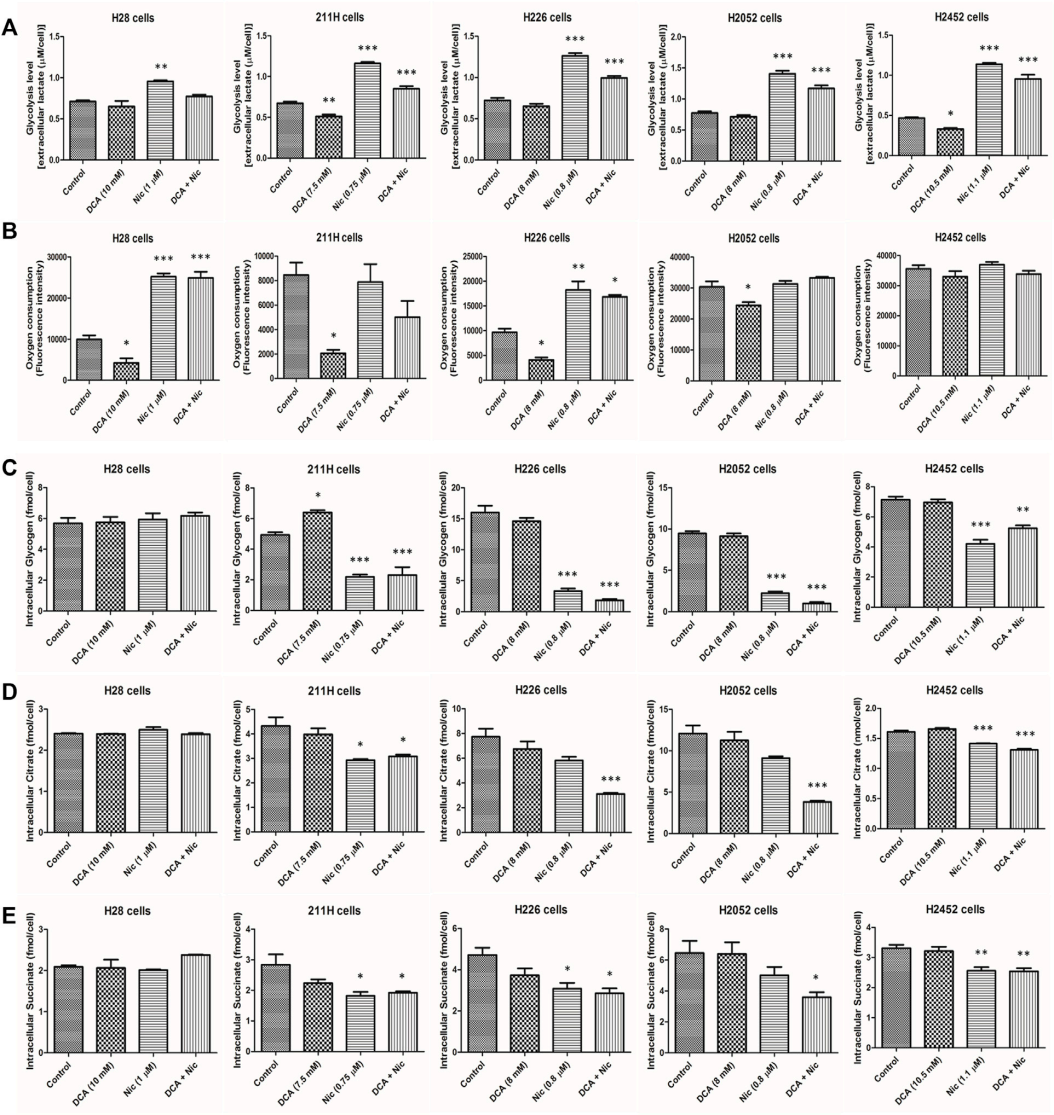
Alteration of glycolysis level, oxygen consumption, intracellular glycogen, citrate and succinate by DCA and/or Nic in MPM cells. **(A)** Nic increased glycolysis rate. **(B)** DCA decreased oxygen consumption in 4 cell lines while Nic and DCA/Nic increased oxygen consumption in 2 cell lines. DCA/Nic decreased intracellular **(C)** glycogen, **(D)** citrate and **(E)** succinate in 4 cell lines.

DCA decreased extracellular lactate in 211H and H2452 cells. On the contrary, Nic elevated extracellular lactate in all cell lines but was partially reversed when combined with DCA (Figure 2A).

#### 3.3.2 Oxygen consumption (oxidative phosphorylation)

DCA decreased oxygen consumption in H28, 211H, H226, and H2052 cells. On the contrary, Nic and DCA/Nic increased oxygen consumption in H28 and H226 cells (Figure 2B).

Interestingly, Nic and DCA/Nic elevated both glycolysis and oxidative phosphorylation in H226 cells.

#### 3.3.3 Intracellular glycogen

DCA increased intracellular glycogen in 211H cells while Nic and DCA/Nic decreased intracellular glycogen in 211H, H226, H2052, and H2452 cells (Figure 2C).

#### 3.3.4 Intracellular citrate

Intracellular citrate was downregulated by Nic and DCA/Nic in 211H and H2452 cells. On the contrary, only DCA/Nic decreased intracellular citrate in H226 and H2052 cells (Figure 2D).

#### 3.3.5 Intracellular succinate

Intracellular succinate concentration was decreased by Nic and DCA/Nic in 211H, H2052, and H2452 cells and decreased by DCA/Nic in H2052 cells (Figure 2E).

#### 3.3.6 Glucose uptake as well as intracellular glucose, pyruvate, lactate and ATP

DCA/Nic neither altered the level nor induced consistent alteration among different cell lines (Supplementary Figure S2).

In general, glycolysis was upregulated while intracellular glycogen, citrate and succinate were downregulated by DCA/Nic, indicating that the Warburg effect was enhanced by DCA/Nic treatment.

### 3.4 Induction of apoptosis

**FIGURE 3:**
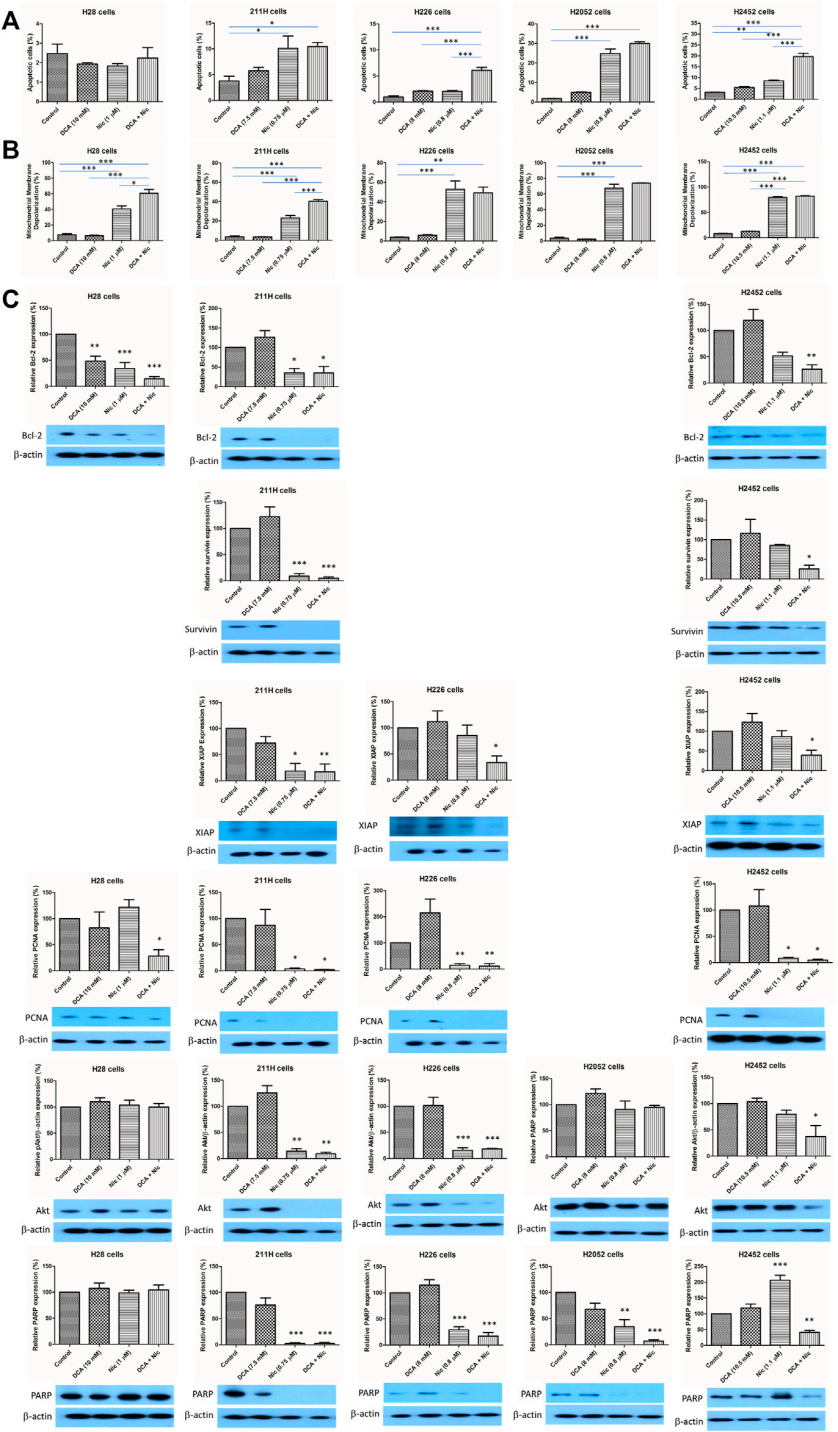
DCA/Nic induced apoptosis in MPM cell lines. DCA/Nic increased **(A)** apoptosis and **(B)** mitochondrial membrane depolarization. **(C)** DCA/Nic downregulated Bcl-2, survivin, XIAP, PCNA, Akt, and PARP in a cell-line specific manner.

In H28 cells, there was no alteration in apoptotic cells by any treatment. In 211H and H2052 cells, Nic and DCA/Nic induced a similar level of apoptosis. In H226 cells, DCA/Nic increased apoptosis. In H2452 cells, Nic elevated the percentage of apoptotic cells and was further enhanced by DCA/Nic (Figure 3A).

### 3.5 Increase in mitochondrial membrane depolarization

Mitochondrial membrane depolarization is another indication of apoptosis. In H28 and 211H cells, Nic increased mitochondrial membrane depolarization and this was further elevated when combined with DCA. In H226, H2052, and H2452 cells, Nic and DCA/Nic induced a similar level of mitochondrial membrane depolarization (Figure 3B).

### 3.6 Alteration of protein expression

Bcl-2, survivin and XIAP are anti-apoptotic proteins. PCNA and Akt are related to cell proliferation while PARP regulates DNA repair. In H28 cells, Bcl-2 was downregulated by DCA, Nic and DCA/Nic while PCNA level was reduced by DCA/Nic. In 211H cells, the expression of Bcl-2, survivin, XIAP, PCNA, Akt and PARP was decreased by Nic and DCA/Nic. In H226 cells, the expression of XIAP was downregulated by DCA/Nic while PCNA, Akt and PARP were suppressed by Nic and DCA/Nic. In H2052 cells, only PARP was downregulated by Nic and DCA/Nic. In H2452 cells, Bcl-2, survivin, XIAP, Akt and PARP were suppressed by DCA/Nic while PCNA expression level was decreased by Nic and DCA/Nic (Figure 3C). DCA/Nic induced apoptosis and inhibited proliferation and DNA repair in MPM cell lines.

### 3.7 Elevation of reactive oxygen species

**FIGURE 4:**
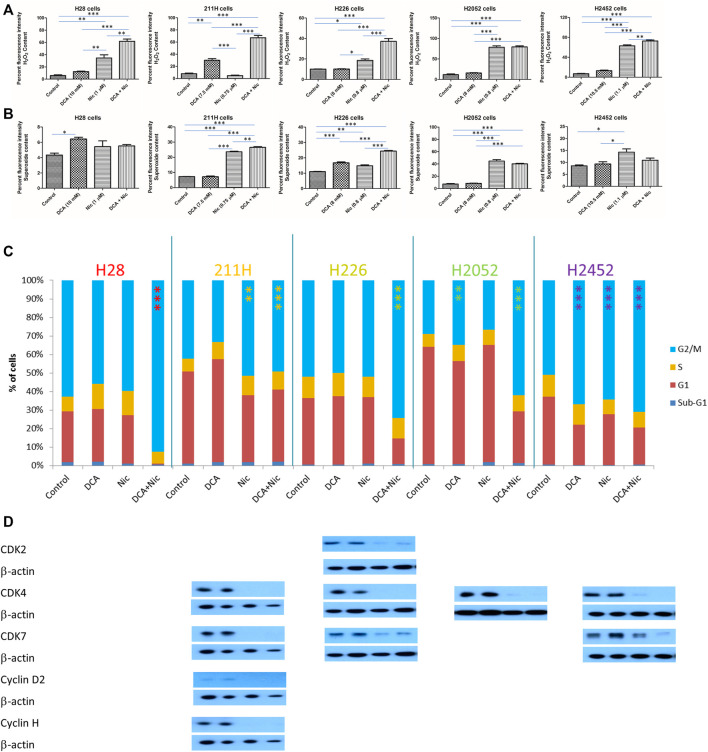
DCA/Nic increased ROS and induced G2/M arrest in MPM cell lines. DCA/Nic elevated **(A)** hydrogen peroxide, **(B)** superoxide and **(C)** G2/M arrest as well as **(D)** downregulating CDK2, CDK7, cyclin D2, and cyclin H in a cell-line specific manner.

In H28, H226, and H2452 cells, Nic increased the hydrogen peroxide level that was further enhanced by addition of DCA. In 211H cells, DCA elevated hydrogen peroxide level that was further increased in DCA/Nic arms. In H2052 cells, hydrogen peroxide was upregulated by Nic and further enhanced in the DCA/Nic group (Figure 4A).

In H28 cells, superoxide was slightly increased by DCA. In 211H cells, Nic elevated the superoxide level that was further enhanced by DCA/Nic. In H226 cells, both DCA and Nic increased superoxide and was further enhanced in the DCA/Nic group. In H2052 cells, Nic and DCA/Nic upregulated the superoxide level to a similar degree. In H2452 cells, Nic slightly increased superoxide content (Figure 4B). DCA/Nic could elevate the level of hydrogen peroxide in all cell lines while superoxide was induced in most of the cell lines.

### 3.8 Induction of G2/M arrest

In H28 and H226 cells, DCA/Nic induced G2/M arrest. In 211H cells, Nic and DCA/Nic caused G2/M arrest. In H2052 cells, DCA induced G/M2 arrest that was further enhanced by DCA/Nic. In H2452 cells, DCA, Nic or DCA/Nic caused G2/M arrest to a similar degree (Figure 4C). In H28 cells, no alteration to different CDKs or cyclins was detected. In 211H cells, CDK4, CDK7, cyclin D2 and cyclin H were downregulated in the Nic and DCA/Nic groups. In H226 cells, CDK2, CDK4, and CDK7 expression was suppressed by Nic and DCA/Nic. In H2052 cells, the expression of CDK4 was decreased in Nic and DCA/Nic arms. In H2452 cells, CDK4, and CDK7 were downregulated by Nic and DCA/Nic (Figure 4D).

G2/M arrest was induced by DCA/Nic in all cell lines and accompanied by downregulation of CDK2, CDK4, cyclin D2, and/or cyclin H.

### 3.9 Inhibition of cell migration

**FIGURE 5:**
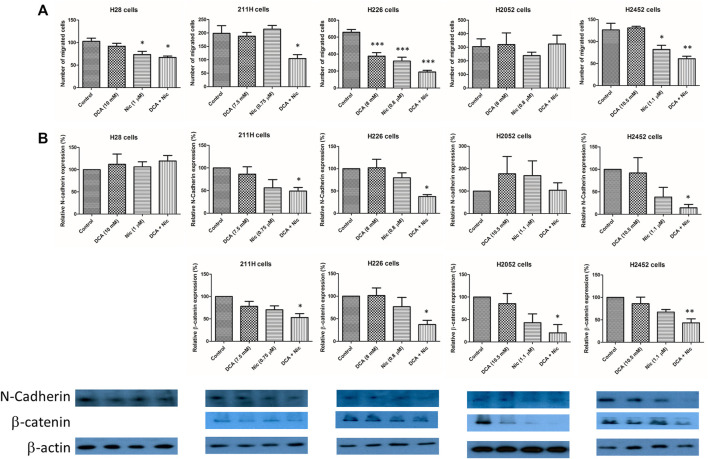
DCA/Nic suppressed the migratory ability of MPM cell lines. **(A)** The number of migrating cells was decreased by DCA/Nic accompanied byh **(B)** suppression of N-cadherin and β-catenin in a cell-line specific manner.

In H28 cells, cell migration was inhibited by Nic and Nic/DCA. In 211H cells, only DCA/Nic supressed cell migration. In H226 cells, cell migratory activity was decreased by both DCA and Nic and further suppressed in the DCA/Nic group. In H2452 cells, cell migration was inhibited by Nic and DCA/Nic (Figure 5A, Supplementary Figure S3). The basal invasive ability of mesothelioma cell lines was low. There was no alteration after different treatments (Supplementary Figure S3). Both N-cadherin and β-catenin were important in cell-cell adhesion. N-cadherin was downregulated by DCA/Nic in 211H, H226, and H2452 cells but unaltered in H28 and H2052 cells. The expression of β-catenin was decreased by DCA/Nic in 211H, H226, H2052, and H2452 cells (Figure 5B).

### 3.10 Tumor suppressive effect of dichloroacetate and niclosamide *in vivo*

**FIGURE 6:**
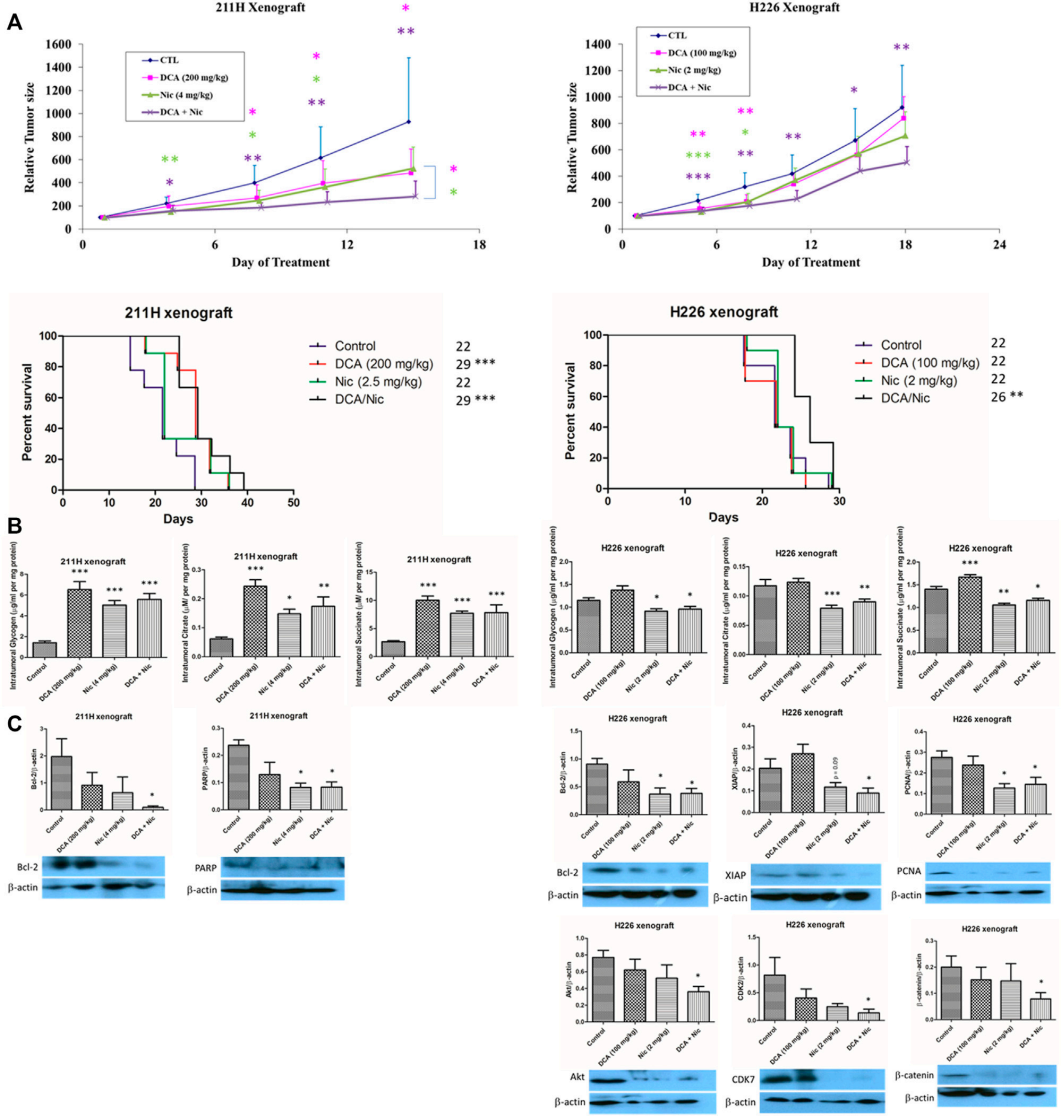
Tumor suppressive effect of DCA/Nic in 211H and H226 xenograft models. **(A)** DCA/Nic inhibited tumor growth and increased median survival in 211H and H226 xenografts. **(B)** Intratumoral glycogen, citrate and succinate were elevated by DCA/Nic in 211H xenografts but reduced in H226 xenografts. **(C)** DCA/Nic downregulated Bcl-2 and PARP in 211H xenografts and Bcl-2, XIAP, PCNA, Akt, CDK7, and β-catenin in H226 xenografts.

In the 211H xenograft, tumor growth was suppressed by a single treatment of DCA or Nic and further repressed by DCA/Nic. The median survival in both DCA and DCA/Nic groups was 29 days, longer than in the control and Nic arms (22 days). In the H226 xenograft, the relative tumor size was reduced in the DCA/Nic group with a consequent longer median survival of 26 days compared with 22 days in the control, DCA and Nic arms (Figure 6A).

Since the intracellular glycogen, citrate and succinate were relatively constantly decreased by treatments *in vitro*, they were tested *in vivo*. In contrast to the *in vitro* results, the intratumoral glycogen, citrate and succinate concentrations were elevated by single and combination treatment in the 211H xenograft. In the H226 xenograft, intratumoral glycogen, citrate and succinate level were decreased in the Nic and DCA/Nic group, consistent with the *in vitro* result (Figure 6B).

In the 211H xenograft, Bcl-2 was downregulated, corroborating the increased apoptosis *in vivo* in the DCA/Nic group. Expression of PARP was suppressed in the DCA/Nic, so the DNA repair mechanism may have been impaired. In the H226 xenograft, downregulation of Bcl-2 and XIAP, PCNA, and Akt, CDK7 and β-catenin by DCA/Nic indicated apoptosis, antiproliferation, G2 arrest and decreased migratory activity respectively (Figure 6C).

## 4 Discussion

Fructose 6-phosphate is converted to fructose 1,6-bisphosphate by phosphofructokinase platelet and is a key step in glycolysis. Pyruvate is converted to lactate by lactate dehydrogenase and is also important in anaerobic glycolysis. Both enzymes play important roles in the Warburg effect. All MPM cell lines in this study showed high basal expression of phosphofructokinase platelet and lactate dehydrogenase: MPM cells exhibited the Warburg effect and were theoretically sensitive to inhibition of such effect. DCA is a pyruvate dehydrogenase kinase inhibitor that shifts glycolysis and lactate production to glucose oxidation in mitochondria. This is known to reverse the Warburg effect (Zhou et al., 2022). Niclosamide is a mitochondrial (oxidative phosphorylation) uncoupler that is known to alter the Warburg effect (Alasadi et al., 2018). The effect of DCA and Nic has not been reported in MPM. This study revealed the combination effect of DCA and Nic in a panel of 5 MPM cell lines with different sub-types (sarcomatoid, biphasic and epithelioid) (Supplementary Figure S4) and 2 xenograft models (Supplementary Figure S5). DCA/Nic disturbed glycolysis and/or oxidative phosphorylation, reduced cell viability and cell migratory activity, induced apoptosis, G2/M arrest and ROS production, and suppressed tumor growth in MPM xenograft models.

DCA has been shown to inhibit cell viability as well as induce apoptosis (Kumar et al., 2012; Duan et al., 2013; Xintaropoulou et al., 2015; Guo et al., 2022a). Niclosamide inhibits cell proliferation and activates apoptosis (Chae et al., 2018; Guo et al., 2022b). In MPM cell lines, DCA alone did not induce apoptosis. On the contrary, Nic alone did induce apoptosis in all MPM cell lines. Interestingly, DCA enhanced Nic-induced apoptosis in 4 cell lines.

PCNA is a proliferative factor that is also involved in DNA replication and repairing (Shen et al., 2021). Akt is important in proliferation and epithelial-mesenchymal transition (Barzegar Behrooz et al., 2022). PARP is essential for DNA repairing and maintenance of telomere integrity (Muoio et al., 2022). Proliferation and DNA repairing were significantly and predominantly inhibited by Nic in MPM cell line models.

Synergism has been reported when DCA is combined with different drugs for anti-cancer research: DCA/cisplatin or gefitnib or elotinib (Al-Azawi et al., 2021), DCA/metformin (Kim et al., 2021; Klose et al., 2021; Korsakova et al., 2021), DCA/3-bromopyruvate (Nikravesh et al., 2021), DCA/sorafenib (Sun et al., 2021), DCA/PX-478 (Parczyk et al., 2021) and DCA/erlotinib (Dyrstad et al., 2021). Said synergistic effect has also been observed when Nic is combined with other agents: Nic/gemcitabine (Guo et al., 2022b), Nic/metformin (Kang et al., 2021) and Nic/doxorubicin (Lohiya and Katti, 2021). We show a novel combination of DCA with Nic that synergistically suppressed tumor growth in MPM cell line and xenograft models. We also tested the combination effect of DCA with metformin or Nic with metformin, but no synergism was observed (data not shown).

DCA has been added to the chemoradiotherapy for locally-advanced head and neck squamous cell carcinoma in a phase II study. Significantly higher response rates have been observed in the DCA arm (Lam et al., 2016). Six myeloma patients have been recruited in a pilot phase II clinical trial and treated with oral clinical grade sodium DCA for 84 days. One patient is maintaining a response to DCA at day 84 and 2 patients showed a partial response at day 28 (Tian et al., 2019). DCA has been combined with anti-parasite drug ivermectin and chemotherapy in 3 cases of malignant tumor with consequent relief of symptoms observed (Ishiguro et al., 2022). The use of DCA is clinically safe and combination regimens containing DCA may increase its efficacy. NIKOLO is a phase II clinical trial of the safety and efficacy of niclosamide in metastatic colorectal cancer patients but results are not yet available (Burock et al., 2018). A phase Ib clinical trial combining an oral bioavailable form of Nic (PDMX1001) with abiraterone and prednisone in castration-resistant prostate cancer patients is underway. Five of 9 patients have more than a 50% prostate-specific antigen response while 2 patients have a complete prostate-specific antigen response (Parikh et al., 2021).

Aerobic glycolysis is preferred by most cancer cells to oxidative phosphorylation for energy production. Aerobic glycolysis and oxidative phosphorylation produce 2 and 36 ATP respectively. Nonetheless the glucose metabolic rate of aerobic glycolysis is 10–100 times faster than complete glucose oxidation in the mitochondria (Shestov et al., 2014). As such, cancer cells express more glucose transporter and increase glucose uptake. This is known as the Warburg effect.

DCA is a pyruvate dehydrogenase kinase inhibitor that suppresses glycolysis (Michelakis et al., 2008; Meng et al., 2020; Guo et al., 2022a) while increasing oxidative phosphorylation (Michelakis et al., 2008; Klose et al., 2021; Belkahla et al., 2022). DCA decreases pyruvate in NSCLC cells (Dyrstad et al., 2021) by promoting pyruvate influx into the TCA cycle (Sharma and Singh, 2020). DCA has been shown to decrease serum pyruvate and lactate in a phase II clinical trial (Lam et al., 2016). DCA decreases lactate level (Michelakis et al., 2008), glucose consumption (Kolesnik et al., 2020; Guo et al., 2022a), lactate production (Dyrstad et al., 2021; Kim et al., 2021) and lactate excretion (Dyrstad et al., 2021), intracellular ATP (Kim et al., 2021) and citrate (El Sayed et al., 2019) but elevates succinate (Zhang et al., 2018).

The effects of Nic in glycolysis, the TCA cycle and oxidative phosphorylation are less well-known. Nic decreases glycolysis (Alasadi et al., 2018; Shangguan et al., 2020). It suppresses oxidative phosphorylation in ovarian cancer (Shangguan et al., 2020) but elevates oxidative phosphorylation in myeloma cells (Khanim et al., 2011). Nic downregulates ATP and lactate (Alasadi et al., 2018).

The effect in the DCA/Nic group was predominantly by Nic in our MPM cell lines: increased glycolysis (Warburg effect) as well as decreased glycogen, citrate and succinate. Although it is well-known that most cancer cells exhibit the Warburg effect and inhibition might cause cell death, enhancement of the Warburg effect above basal level might also induce killing of cancer cells. It has been shown that glycolysis was enhanced when breast cancer cells were treated with a combination of tamoxifen and dasatinib by disabling the use of their fuel supply (Martinez-Outschoorn et al., 2011). In this study, the Warburg effect was downregulated by DCA but upregulated by Nic after 4 h of treatment. In addition, cell viability was reduced and apoptosis was induced after 72 h treatment. It seems that the Warburg effect is well-balanced in MPM cells and either inhibition of the effect by DCA or enhancement by Nic may kill MPM cells.

DCA has been shown to elevate hydrogen peroxide (Xie et al., 2011) and ROS (Kumar et al., 2012; Duan et al., 2013; Kim et al., 2021; Klose et al., 2021; Nikravesh et al., 2021). In addition, ROS is increased when DCA is combined with sorafenib (Sun et al., 2021) or PX-478 (Parczyk et al., 2021). Nic also increases ROS (Zhou et al., 2017; Shangguan et al., 2020; Kaushal et al., 2021; Lohiya and Katti, 2021). In this study, DCA alone increased only hydrogen peroxide in 211H cells and superoxide in H226 cells. On the other hand, Nic elevated hydrogen peroxide in 4 MPM cell lines and superoxide in 3 MPM cell lines. Interestingly, DCA/Nic further upregulated hydrogen peroxide in 4 MPM cell lines and superoxide in 2 MPM cell lines. In general, DCA/Nic increased ROS in MPM cell lines.

DCA induces G1 arrest (Lin et al., 2014; Allende-Vega et al., 2015) and G2/M arrest (Lin et al., 2014; Shen et al., 2015a). G2/M arrest is enhanced by different DCA combinations (Shen et al., 2015b; Zhang et al., 2015; Dong et al., 2016; Verma et al., 2019). Nic induces G0/G1 arrest (Ren et al., 2010; Wu et al., 2020; Kaushal et al., 2021; Lohiya and Katti, 2021), G1 arrest (Wieland et al., 2013; Satoh et al., 2016; Li et al., 2017b; Han et al., 2018; Lee et al., 2020), G1/S arrest (Chae et al., 2018) and G2/M arrest (Li et al., 2015). In this study, G2/M arrest was induced by DCA in H2052 and H2452 cells and by Nic in 211H and H2452 cells. More importantly, DCA/Nic induced G2/M arrest in all MPM cells. Nic predominantly suppressed the expression of different CDKs and cyclins in a cell-line specific manner. Downregulation of CDK2 (Li et al., 2019), CDK4 (Abaza et al., 2015; Li et al., 2019), CDK7 (Abaza et al., 2015), cyclin D2 (Yang et al., 2018) and cyclin H (Dogan Sigva et al., 2019) are related to G2/M arrest. It has been shown that Nic induces downregulation of CDK2 and CDK4 (Li et al., 2017b), but not CDK7, cyclin D2 or cyclin H in other cancer types.

It has been demonstrated that DCA inhibits migration (Florio et al., 2018; Tataranni et al., 2019; Guo et al., 2022a) of cells as well as invasion (Guo et al., 2022a). Nic has been shown to have anti-migratory and anti-invasive properties (Zhu et al., 2019; Guo et al., 2022b; Yeh et al., 2022). In this study, DCA inhibited migration in H226 cells while Nic suppressed migration in 3 MPM cells lines. DCA/Nic synergistically decreased the migratory ability of 211H cells. The action mechanism is partially mediated by downregulation of N-cadherin and β-catenin by DCA/Nic.

The tumor suppressive effect of DCA or Nic combination regimens has been demonstrated in xenografts of different cancer types: DCA/activated natural killer cells/anti-CD20 (Belkahla et al., 2022), DCA/4‐methylumbelliferone (Twarock et al., 2019), DCA/arginase (Verma et al., 2019), DCA/sirtinol (SIRT2 inhibitor) (Ma et al., 2018), DCA/cisplatin (Woolbright et al., 2018), Nic/cisplatin (Liu et al., 2021) and Nic/paclitaxel (Chen et al., 2017). Although either DCA or Nic may exert its tumor suppressive effect in different cancer xenografts, a better anti-cancer effect is usually observed when DCA or Nic is combined with another therapeutic drug. In our MPM xenograft models, a combination of DCA and Nic showed a better initial tumor suppressive response in both MPM xenograft models and increased median survival in H226 xenografts. Since intracellular glycogen, succinate and citrate were relatively consistent reduced by treatment *in vitro*, their levels were investigated *in vivo.* The glycolysis assay kit measured extracellular lactate and was not applicable in xenograft models. To the best of our knowledge, the *in vivo* effect of DCA or Nic on intratumoral glycogen, succinate and citrate has not been reported.

Cells with a lower glycolysis rate increase conversion of glucose to glycogen and glycogen storage (also known as glycogenesis). Increase in glycogen storage indicates that cancer cells shift away from cancer metabolism including the Warburg effect and glycolysis. On the other hand, oxidative phosphorylation is decreased when cellular glycogen storage declines. Furthermore, rates of proliferation and division are slowed when cells store more glycogen (Steenbergen et al., 2018). Accumulation of citrate is a sign of inhibition of glycolysis. When glycolysis is inhibited, the energy and building blocks are decreased, so proliferation is suppressed. Overproduction of citrate and ATP inhibits cell proliferation. Elevation of oxidative phosphorylation increases cellular ATP and citrate levels as well as ROS (Philippe IS et al., 2017). Succinate is a tumor promoter and also a marker of hypoxia and mitochondrial dysfunction. Accumulation of cellular succinate increases production of ROS and DNA hypermethylation (Ristic et al., 2017).

In the 211H xenograft, intratumoral glycogen, succinate and citrate were upregulated in DCA, Nic and DCA/Nic arms, contrary to *in vitro* results. Although the results are paradoxical, the *in vivo* data are more favorable since they more closely mimic the human system. Treatments inhibited glycolysis/Warburg effect (increase in glycogen) and proliferation while increasing oxidative phosphorylation (increase in glycogen) and ROS. In accordance, glycogen, succinate and citrate were reduced by DCA/Nic in both H226 cell lines and nude mice model: glycogen was decreased by treatments in the H226 xenograft, indicating that the Warburg effect was enhanced while oxidative phosphorylation was decreased. As such, DCA/Nic disturbed glycolysis/oxidative phosphorylation and suppressed tumor growth *in vivo*. In the 211H xenograft, downregulation of Bcl-2 and PARP by DCA/Nic indicated activation of apoptosis and suppression of DNA repairing activity. In the H226 xenograft, tumor growth was suppressed by DCA/Nic and partially mediated by apoptosis (downregulation of Bcl-2 and XIAP), anti-proliferation (decrease in expression of PCNA and Akt), G2/M arrest (CDK7 downregulation) and suppressed migration (decline in expression β-catenin). Downregulation of Bcl-2 (Liu et al., 2021) and β-catenin (Guo et al., 2022b) by Nic *in vivo* has been reported. Furthermore, lower expression of CDK7, AKT1, PARP1 (*p* < 0.1), CCND2 (cyclin D2), CDK2, CDK4, BIRC5 (survivin), PCNA and CDH2 (N-cadherin) (*p* < 0.005) have been shown to result in longer median survival of patients with mesothelioma (Supplementary Figure S6). They are downregulated *in vitro* and some of them are suppressed *in vivo* by DCA/Nic. This may partially explain the potent anticancer effect *in vitro* and prolonged median survival *in vivo.*

## 5 Conclusion

In summary, DCA/Nic synergistically inhibited different subtypes of MPM *in vitro* and *in vivo,* partially mediated by disturbed glycolysis/oxidative phosphorylation, activation of apoptosis, anti-proliferation, induction of G2/M arrest, production of ROS and/or inhibition of migration.

## Data Availability

The original contributions presented in the study are included in the article/Supplementary Material, further inquiries can be directed to the corresponding author.
