# Supplementary figures and images for: Disturbance of the Warburg effect by dichloroacetate and niclosamide suppresses the growth of different sub-types of malignant pleural mesothelioma in vitro and in vivo
Source: Front Pharmacol. 2022 Oct 11;13:1020343. doi: 10.3389/fphar.2022.1020343 (PMC9592830; doi:10.3389/fphar.2022.1020343)

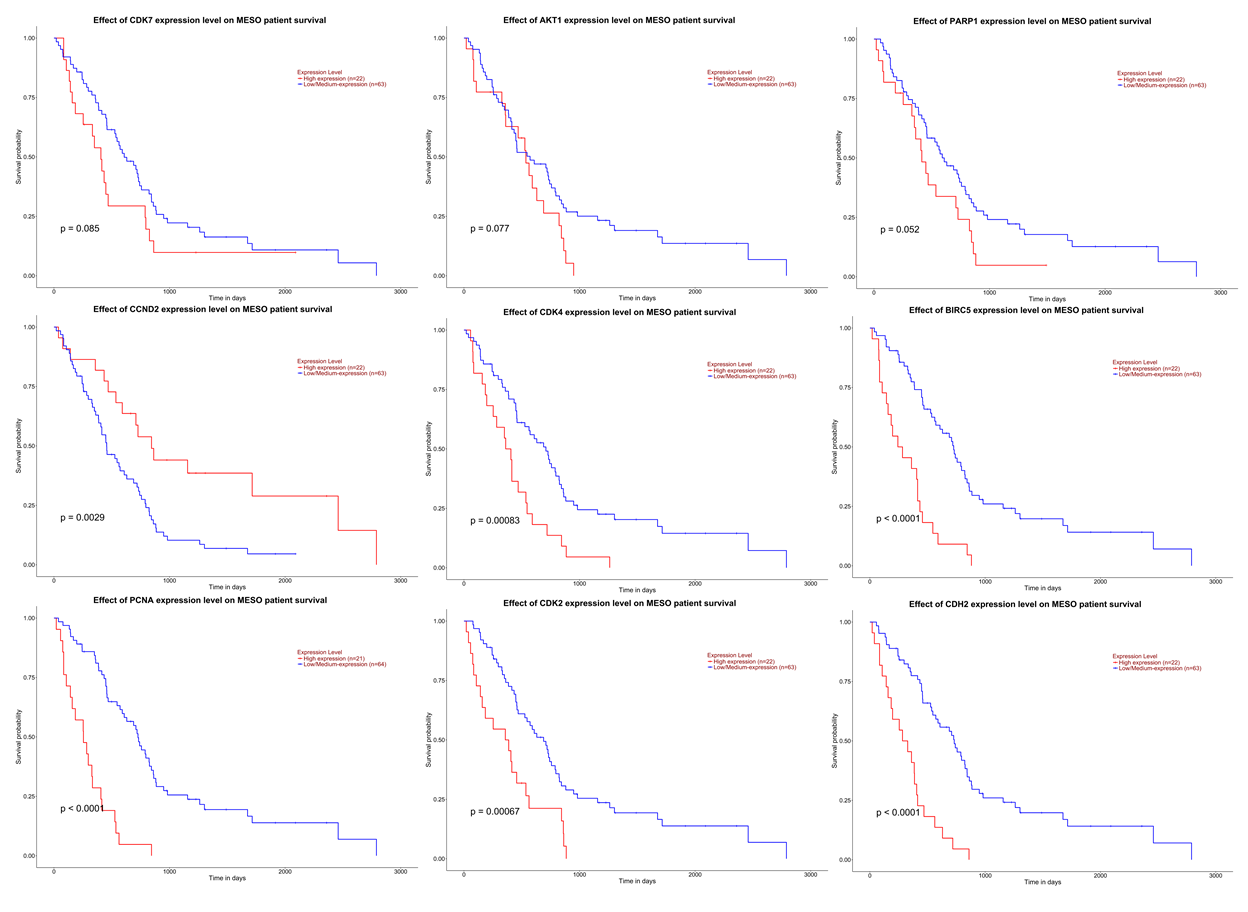

Supplement: Supplementary file 1 [file Image6.TIF]

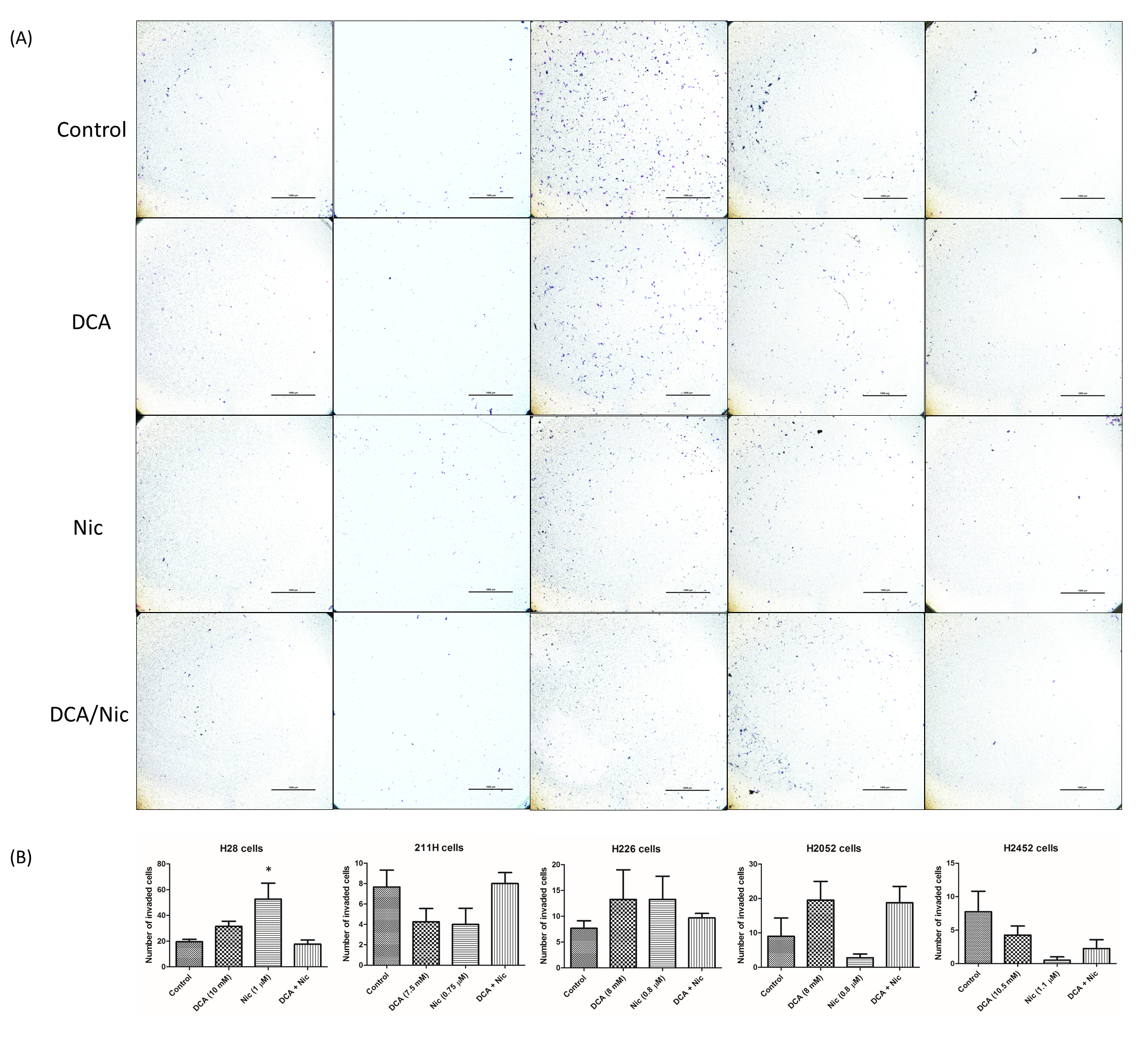

Supplement: Supplementary file 3 [file Image3.TIF]

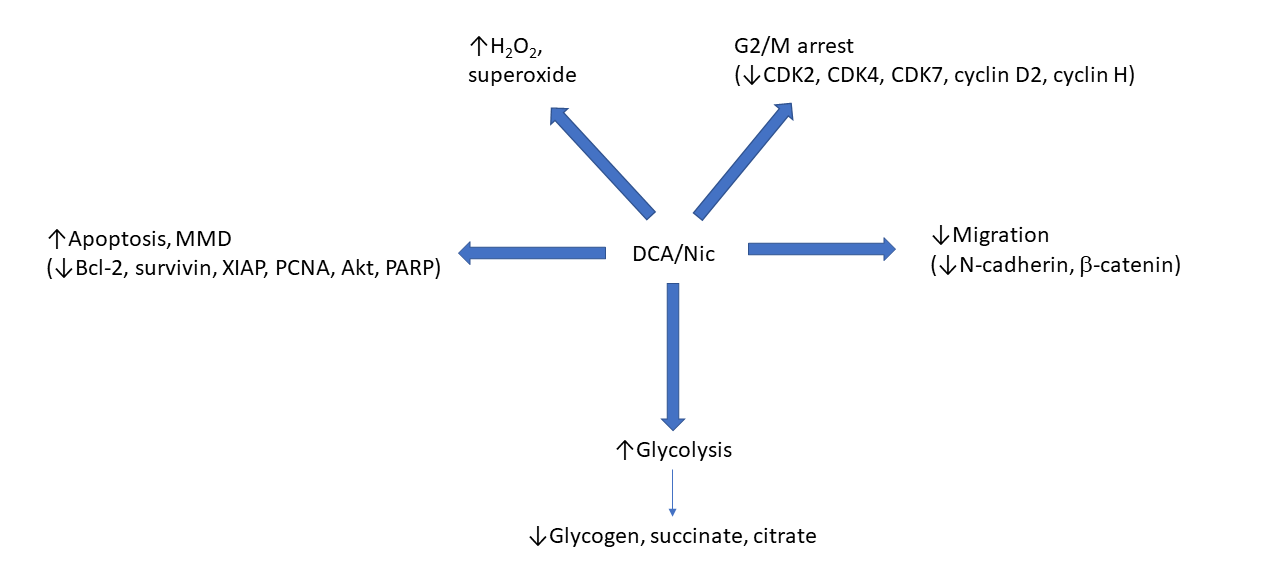

Supplement: Supplementary file 4 [file Image4.TIF]

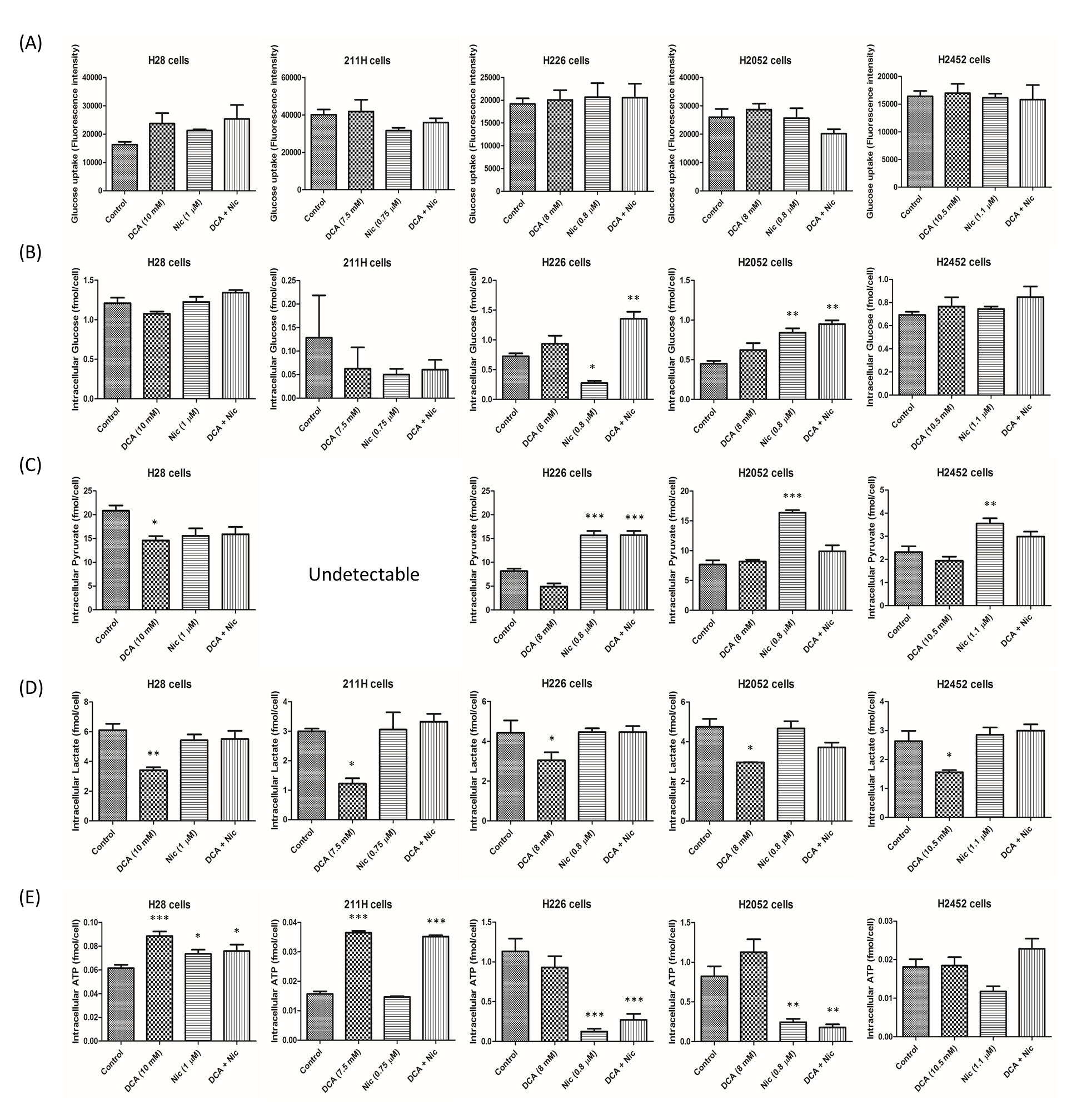

Supplement: Supplementary file 5 [file Image2.TIF]

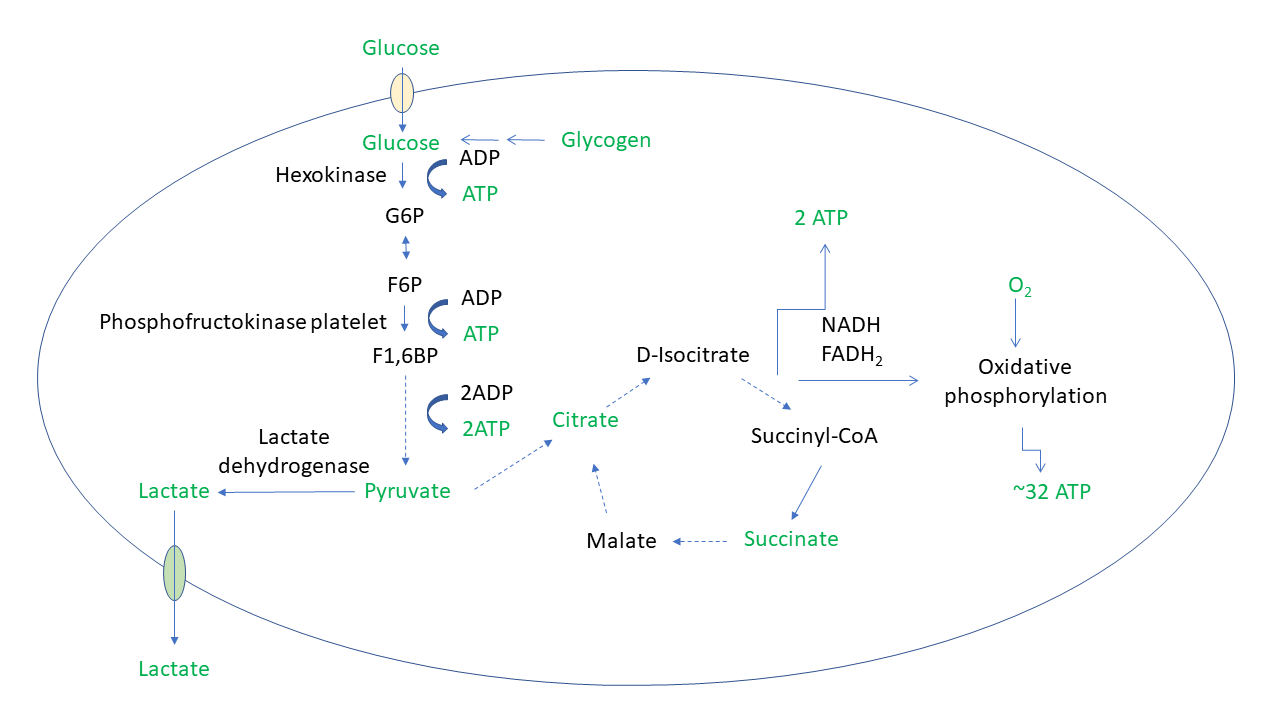

Supplement: Supplementary file 6 [file Image1.TIF]

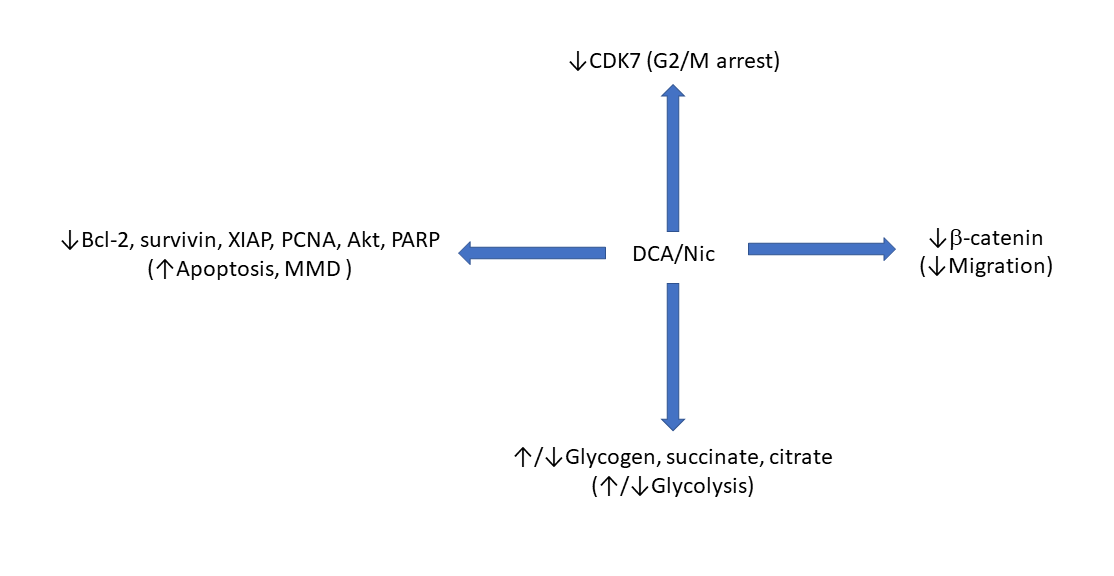

Supplement: Supplementary file 7 [file Image5.TIF]
