# Supplementary material for: Disturbance of the Warburg effect by dichloroacetate and niclosamide suppresses the growth of different sub-types of malignant pleural mesothelioma in vitro and in vivo
Source: Front Pharmacol. 2022 Oct 11;13:1020343. doi: 10.3389/fphar.2022.1020343 (PMC9592830; doi:10.3389/fphar.2022.1020343)
Supplement: Supplementary file 2 [file Table1.DOCX]

Supplementary Table 1. The IC_50_ values of DCA, Nic and DCA/Nic treated cells and the combination effect of DCA/Nic in a panel of MPM cell lines.

|  |  | **H28** | **211H** | **H226** | **H2052** | **H2452** |
| --- | --- | --- | --- | --- | --- | --- |
| **DCA (mM)** | Single drug | 20 | 21 | 20 | 23 | 25 |
| **Niclosamide (μM)** |  | 2 | 2 | 3 | 3 | 2.5 |
| **DCA (mM)** | Comb | 10 | 7.5 | 8 | 8 | 10.5 |
| **Niclosamide (μM)** |  | 1 | 0.75 | 0.8 | 0.8 | 1.1 |
| **Combination effect** | Comb | synergism to strong synergism | moderate synergism to synergism | moderate synergism to synergism | moderate synergism to additive | moderate synergism to slightly antagonism |
